# Multi-Isotope Internal Standardization for Inductively Coupled Plasma Mass Spectrometry

**DOI:** 10.1021/acsomega.5c07066

**Published:** 2025-11-14

**Authors:** Micah X. DeCourcey, Abigail J. Crossman, Willis B. Jones

**Affiliations:** Department of Chemistry and Biochemistry, 4127 University of North Florida, Jacksonville, Florida 32224, USA

## Abstract

Recent developments in trace analyte determination have evolved from the traditional relationship relating analyte signal to concentration to “multisignal” methods where both axes are measured instrumental signals. However, many multisignal methods are limited in some fashion: Some have low sample throughput, some result in poor accuracies in difficult sample matrices, and others require several significant signals for each analyte species. Multi-isotope internal standardization (MIIS) is a newly developed calibration method for inductively coupled plasma mass spectrometry (ICP-MS) that addresses the issues presented by other multisignal techniques. Multiple isotope masses for both the analyte species and several internal standards are used to build a calibration curve. Only two solutions are required to perform the calculation. Spike recovery experiments performed in complex matrices known to be problematic for ICP-MS yielded analyte recoveries of approximately 100% with relative standard deviations on the order of a few percent. MIIS was found to outperform both traditional calibration techniques as well as other multisignal methods. MIIS was validated through the determination of a suite of analytes in three certified reference materials with recoveries ranging from 85 to 111%. MIIS provides a clear advancement over other multisignal calibration techniques developed for ICP-MS, resulting in an exceptionally high number of calibration points even though only two calibration solutions are prepared.

## Graphical abstract

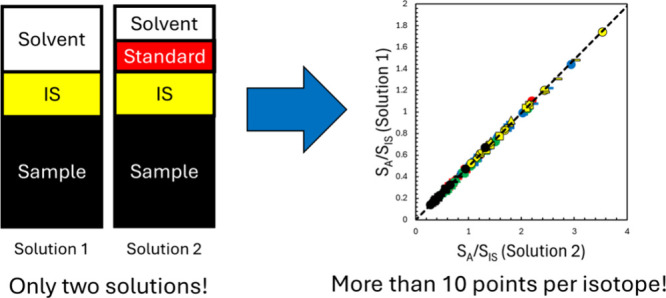

## 1 Introduction

The primary objective of any quantitative analytical calibration is to determine accurate and precise concentrations for all analytes of interest within a given sample. However, such measurements are often constrained by the chemical complexity of the sample matrix, by minor variations in instrumental operating conditions that occur during an analysis, or by some combination of the two. Most calibration strategies establish a direct relationship between the concentration of an analyte in solution and its corresponding signal measured during an experiment. External standard calibration (EC) is a widely used calibration technique that involves the preparation of a series of standards containing known concentrations of the analyte in blank solvent. The analyte signal measured for each standard is then related to the known, prepared concentration of the standards, and the resulting calibration is used for the determination of the analyte in the unknown samples. Although straightforward to implement, EC on its own does not address variability introduced by instrumental drift or sample-specific matrix effects and thus frequently yields results of poor accuracy and reliability.^1^

Correction of signal variations caused by the chemical makeup of a sample is a daunting task. The sample matrix, defined as all substances in the sample other than the analytes, drastically impacts the reliability of analytical calibrations if left unaddressed. These “matrix effects” cause identical concentrations of an analyte across samples with differing compositions to produce markedly different signal intensities, thereby invalidating any quantitative measurement. Matrix-matched calibration (MMC) is a variation of EC in which the calibration standards are prepared in a matrix mimicking the expected sample matrix, resulting in a correction of matrix effects.^1–5^ This is often achieved through the addition of certified reference materials to the standards or by purposeful addition of expected sample concomitants. However, MMC is not a straightforward process, requiring significant upfront knowledge of sample composition for success. Thus, the most frequently utilized method for matrix effect correction is standard addition (SA). In SA, the sample is divided equally into several portions with increasing amounts of analyte standard added to each. As each prepared standard contains the same amount of the unknown sample matrix, SA provides an exceptionally effective means of correcting for all matrix effects. However, the technique has notable drawbacks, as each individual sample requires its own full calibration curve, which greatly reduces sample throughput, and the resulting data can be complex to process and interpret.^6,7^

In contrast to sample matrix effects, the correction of fluctuations in instrumental parameters is relatively simple. For EC determinations, the use of periodic quality control checks can address instrumental drift over time. However, internal standardization (IS) remains the most commonly used approach for mitigating variations in instrumental conditions during the analysis. The method entails introducing a distinct species (known as the internal standard) into all standards and samples prior to the analysis. Calibration is then based on the signal ratio of the analyte to the internal standard. The effectiveness of IS depends on several key assumptions: The internal standard must not be present in the samples, must not have any interferences with the analyte, and must be subject to the same instrumental fluctuations and respond to changes in a similar fashion as the analyte.^8,9^ If these three assumptions are true, then using the signal ratio to build the calibration curve corrects for instrumental fluctuations such as gas flow rates and rates of sample introduction, among others.^1,10,11^ In practical applications, these assumptions are often violated, especially when the composition of the sample matrix diverges from that of the calibration standards. In inductively coupled plasma mass spectrometry (ICP-MS) for example, variations in plasma temperature, shifts in ionization efficiency, and changes in signal intensity over the course of an analysis can compromise measurement accuracy if they are not adequately accounted for.^8,9^ In the end, the success of internal standardization is reliant on the selection of the internal standard species, which is particularly difficult when multiple analytes are of interest.^12^ Even if the properties of the chosen internal standard closely match those of the analyte, there is no guarantee that the internal standard is the optimal selection.^13–15^

Overall, these limitations highlight the need for more resilient calibration strategies that can reliably correct for both physical and chemical sources of analytical error. Recent innovations in trace-level calibration methodology can be categorized as “multisignal” methods, where both axes of prepared calibration curves are measured signals in contrast to the traditional relationship of signal to concentration. Information regarding the analyte concentration in each sample is typically extracted from the calculated slopes and intercept values of these plots. The difficulties encountered in selecting suitable internal standards are often alleviated through the application of these methods, many of which also involve matrix-matched sample preparation that inherently addresses sample matrix effects.^16^

Standard dilution analysis (SDA) is a matrix-matched calibration technique that utilizes automated dilution of an analyte standard as solution flows through tubing from the autosampler to the instrument during a measurement.^17–20^ The monitored analyte signals are plotted against two different internal standards that provide a measure of how much standard is present in the solution stream at any given point in time. SDA is the most widely employed multisignal calibration method proposed to date due to its similarity to conventional calibration methods. Applications of SDA have been exhibited using flame atomic emission spectrometry (FAES),^21^ flame atomic absorption spectrometry (FAAS),^22^ inductively coupled plasma optical emission spectrometry (ICP-OES),^17–20,17–20,23–26^ microwave-induced plasma optical emission spectrometry (MIP-OES),^27,28^ visible absorption spectrometry,^17^ Raman spectroscopy,^29^ and ICP-MS.^30^ However, the method was initially largely limited to instruments capable of capturing all relevant signals simultaneously, making it impractical for sequentially measuring instrumentation such as typical ICP-MS. The single literature example of using SDA as originally proposed for ICP-MS proved the technique could work, but it was limited in the number of analytes that could be simultaneously determined without the calibration failing.^30^ The more recent development of multichannel dilution analysis (MCDA) broadened SDA’s applicability to instruments that operate sequentially, overcoming the need for simultaneous determination at the cost of lowering sample throughput.^31,32^ All SDA-based strategies perform online dilutions to generate the calibration points, removing the need for preparing multiple standard solutions and correspondingly improving sample throughput when compared to traditional standard additions. In SDA-type methods, the observed change in signal continues to result from a physical change in the concentration of the analyte, much like that in conventional calibration techniques.

Other novel multisignal calibration strategies depart significantly from traditional methods, altering the nature of the signal being measured instead of concentration to generate the signal change necessary for calibration. Among these unconventional approaches, the most widely adopted to date is multienergy calibration (MEC), a matrix-matched method that constructs a calibration curve by measuring all analytes of interest at different characteristic emission or absorption wavelengths.^33^ While largely successful for the species that it can quantify, MEC was inherently limited in that it requires the analyte species to have several strong characteristic wavelengths, rendering it impractical for many essential analytes. The theory behind MEC was adapted specifically for use with ICP-MS in a technique called multi-isotope calibration (MICal), which constructs calibration curves by simultaneously measuring multiple isotopes of the same analyte.^34^ MICal using ICP-MS has proven successful for the determination of many essential trace analytes in a wide variety of complex sample matrices.^35–39^ MICal suffers from the same limitation as MEC, in that it requires the analyte of interest to have several isotopes in order to construct a calibration curve. A unique twist on the MICal method called multispecies calibration (MSC) utilized tandem ICP-MS/MS to react analyte species with gases in a collision cell to form additional molecular species that could be utilized for the calibration.^40^ MSC overcame some of the multi-isotope limitations of MICal but required the use of more complex triple quadrupole ICP-MS instruments, as well as analytes that readily react.

Multi-internal standard calibration (MISC) is another multisignal technique that is used to determine analyte concentration by normalizing the signal of a single analyte to multiple internal standard species simultaneously. This eliminates the need to identify an optimal internal standard for each analyte, as the combined use of several internal standards generates a more comprehensive correction that better reduces signal variability.^41^ Although only recently described, MISC has been demonstrated successfully using both ICP-OES and ICP-MS analyses.^41,42^ However, a key limitation of the MISC method is its inability to compensate for severe matrix effects, resulting in accuracy levels comparable to those achieved through conventional external calibration and internal standardization when applied to complex samples.

Isotope dilution mass spectrometry (IDMS) is a more well-known calibration technique used for the accurate determination of trace analytes using ICP-MS. In IDMS, a known amount of an isotopically enriched analyte standard is added to a sample, and the measured isotopic ratio is used to determine the concentration of the trace analyte in the unknown.^43,44^ IDMS offers highly accurate and precise trace analyte measurements using ICP-MS.^45,46^ While the method is largely considered to be one of the best for measurement accuracy and precision using ICP-MS, it is not without drawbacks. Notably, IDMS requires the use of an isotopically enriched analyte standard. Additionally, the method requires the measurement of multiple isotopes without isobaric interference, which can prove troublesome in certain analyses such as the determination of rare earth elements^44^ and makes IDMS inapplicable for the determination of elements with only a single isotope.

Recent developments in the MEC and MISC techniques using ICP-OES resulted in a method called multiwavelength internal standardization (MWIS), which is essentially a fusion of the two calibration strategies that addressed some of the weaknesses of both.^47^ MWIS utilized several emission wavelengths for analytes (if they were available) and normalized each to several emission wavelengths for a series of internal standards, resulting in a high number of signal ratios used for the calibration. MWIS allowed for the determination of the critical toxic analytes As, Cd, and Pb, which was not possible using MEC alone at the trace level due to the limited number of suitable emission wavelengths for each. In addition, the sample preparation corrected for sample matrix effects and did not require internal standard optimization due to the high number of IS species selected, effectively minimizing the weaknesses of MISC.

Multi-isotope internal standardization (MIIS) is a novel, matrix-matched, multisignal calibration method first presented here that was developed to address the direct limitations of the MICal, MSC, and MISC techniques as applied to ICP-MS. The core of the MIIS approach is the use of multiple isotopes for each analyte of interest (where available), normalized using multiple isotopes of a series of internal standards. This configuration produces a large number of signal ratios for use in the calibration curve, even though only a single concentration of the added standard is introduced during measurement. The method also addresses some of the limitations of IDMS calibration, as standards of natural isotopic abundance can be utilized, and MIIS can be used to determine trace analytes with only one isotope. A comprehensive description of the required solution preparation and a theoretical derivation of the MIIS approach are provided below.

### MIIS Solution Preparation and Theoretical Derivation

The solution preparation required for MIIS resembles that used for several related multisignal calibration techniques.^17,20,33,34,40,47^ MIIS requires the preparation of only two solutions to construct a complete matrix-matched calibration curve: Solution 1 was composed of 50% of the sample solution of interest and 50% of a suite of internal standards and blank solvent, with solution 2 containing 50% of the same sample solution and 50% of the same amount of the same IS species added to solution 1, an aliquot of a standard solution containing all analytes of interest at known concentration, and sufficient blank solvent to maintain identical sample matrix conditions as solution 1. Schematics depicting the solution preparation are provided in Figure 1.

**Figure 1:**
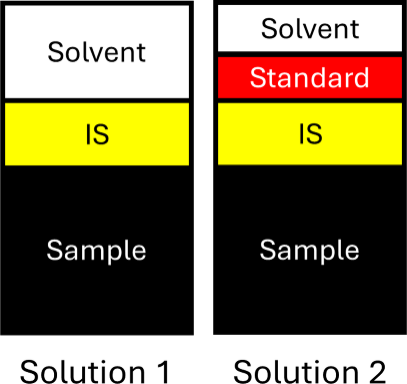
Schematic detailing the required solution preparation for calibration by MIIS.

A brief mathematical derivation of MIIS follows, mimicking that of MWIS,^47^ and essentially incorporating the use of internal standard species into the MEC and MICal calculations.^33,34^ In general, the signal of an analyte (*S*) observed in a measurement is equal to the sensitivity (*m*) for the analyte on the instrument being utilized multiplied by the analyte concentration (*C*). For example, consider an analyte in solution 1: The measured signal (*S*_A1_) is given by eq 1, where the concentration in solution 1 (*C*_A1_) is equal to the concentration of the analyte in the sample (*C*_sam_). The same theory can be applied for solution 2 as shown in eq 2, with the difference being that the concentration of analyte (*C*_A2_) has two components as the analyte is present in both the sample (*C*_sam_) and the standard (*C*_std_). A similar relationship holds for the internal standards, which are present at the same concentration in both solutions 1 and 2 (thus the measured signal should be identical for each), as shown in eq 3.

$$S_{A1}=m_{A}C_{A1}=m_{A}C_{\mathrm{sam}}$$ 1

$$S_{A2}=m_{A}C_{A2}=m_{A}(C_{\mathrm{sam}}+C_{\mathrm{std}})$$ 2

$$S_{\mathrm{IS}}=S_{\mathrm{IS}1}=S_{\mathrm{IS}2}=m_{\mathrm{IS}}C_{\mathrm{IS}}$$ 3

The internal standards in MIIS are used in the same fashion as traditional internal standards and in MISC: Analyte signals measured in solutions 1 and 2 are divided by the internal standard signal measured in the same solution. These ratios for solutions 1 and 2 are shown in eqs 4 and 5, respectively, where *n* denotes all of the monitored internal standard species.

$$\frac{S_{A1}}{S_{\mathrm{IS}1(n)}}=\frac{m_{A}}{m_{\mathrm{IS}(n)}}\times \frac{C_{\mathrm{sam}}}{C_{\mathrm{IS}(n)}}$$ 4

$$\frac{S_{A2}}{S_{IS2\left(n\right)}}=\frac{m_{A}}{m_{IS\left(n\right)}}\times \frac{\left(C_{sam}+C_{std}\right)}{C_{IS\left(n\right)}}$$ 5

The sensitivity ratios (*m*_A_/*m*_IS_) for the two solutions are equal as the two solutions are matrix-matched because they hold an identical amount of the sample. Therefore, the individual species sensitivity terms for each species (and isotope) monitored during the MIIS measurement cancels in the calculation. This is a direct improvement over the previous descriptions of MISC, which assumed constant sensitivity ratios even though the sample and standard solutions were prepared separately, leading to relatively poor results for complex sample matrices.^41^ The fact that the sensitivity ratios are equal allows for eqs 4 and 5 to be combined and rearranged as shown in eqs 6 and 7, with eq 7 providing a linear relationship when the ratios measured in solution 1 are plotted against the ratios measured in solution 2. The slope of this linear relationship contains the concentration of the analyte in the sample (*C*_sam_), which is the intended result of the measurement. Thus, *C*_sam_ is calculated using a simple algebraic rearrangement of the determined slope and the known concentration of the analyte standard added to solution 2, as shown in eq 8.

$$\frac{S_{A1}}{S_{\mathrm{IS}1(n)}}\times \frac{C_{\mathrm{IS}(n)}}{C_{\mathrm{sam}}}=\frac{S_{A2}}{S_{\mathrm{IS}2(n)}}\times \frac{C_{\mathrm{IS}(n)}}{C_{\mathrm{sam}}+C_{\mathrm{std}}}$$ 6

$$\frac{S_{A1}}{S_{\mathrm{IS}1(n)}}=\frac{C_{\mathrm{sam}}}{C_{\mathrm{sam}}+C_{\mathrm{std}}}\times \frac{S_{A2}}{S_{\mathrm{IS}2(n)}}$$ 7

$$C_{\mathrm{sam}}=\frac{\mathrm{slope}}{1-\mathrm{slope}}\times C_{\mathrm{std}}$$ 8

The inclusion of multiple internal standard species enables the construction of numerous calibration points from a single monitored analyte, akin to MISC.^41,42^ Simultaneous measurement of multiple isotopes for selected analytes (if possible) and all of the internal standards further increase the number ratios that can be used to perform the calibration. For instance, consider an MIIS measurement performed for the quantification of an analyte that only has two naturally occurring isotopes. Suppose the solutions are spiked with three internal standard elements, each of which has three isotopes. If only one of the analyte isotopes is used in the calculation, there are still nine signal ratios available for constructing the calibration curve, as the single analyte signal can be correlated with the nine distinct internal standard isotopes. Incorporating the second analyte isotope into the measurement doubles the number of calibration points to 18. Because each of the isotope-specific sensitivity factors cancel out during the calculation for the prepared solutions 1 and 2, any number of analyte and internal standard isotopes can be incorporated into the measurement, and all of the resulting ratios will align onto a single calibration curve, provided that there are no significant isobaric interferences. This strategy of combining multiple internal standards and isotopes to derive many signal ratios from potentially a single analyte isotope overcomes the primary limitation of MICal and IDMS, which are restricted to elements with several isotopes of significant natural abundances.^34,43–46^ The proposed MIIS strategy also directly overcomes the major limitation of MSC, as the technique is applicable to single quadrupole ICP-MS and does not require analytes to readily interact with intermediate reaction gases.^40^

The preliminary MIIS measurements conducted and presented in this work were designed to evaluate several key objectives. A suite of target analytes (largely having only one or two significant naturally occurring isotopes) were selected for proof of concept. Limits of detection for each analyte and a suggested working range for MIIS were determined, and the method was validated using certified reference materials (CRMs). Spike recovery experiments at the lower end of the established working range were carried out in sample matrices known to present challenges for direct ICP-MS analysis in order to assess the method’s ability to correct for matrix effects. MIIS results were compared to those obtained using EC, IS, and SA methods as well as MISC and MICal. Additional discussion around the selection of appropriate internal standard isotopes and considerations for successful implementation of the MIIS technique is also included.

## 2 Materials and Methods

### Standards and Sample Preparation

All solutions were prepared from individual 10.00 mg L^–1^ trace metal standards obtained from High Purity Standards (HPS) (North Charleston, SC, USA) in a final volume of 1% (v/v) trace metal grade HNO_3_. A single analyte stock consisting of 50.0 μg L^–1^ Li, Be, V, Mn, Co, Ag, Cd, Tl, Pb, and U was prepared for proof of concept and spike recovery experiments in simulated complex sample matrices and was used to prepare the sample portion of both solutions 1 and 2 as well as the standard portion of solution 2. A secondary analyte stock solution containing 50.0 μg L^–1^ V, Cr, Mn, Co, Sr, Mo, Ag, Cd, and U was prepared as the standard for use in the analysis of certified reference materials (CRMs). An internal standard stock solution containing 50.0 μg L^–1^ Nd, Gd, Dy, Er, and Yb was prepared for incorporation into both solutions 1 and 2 for all MIIS measurements. All replicate preparations of solutions 1 and 2 for all of the experiments detailed throughout this manuscript had final volumes of 10.00 mL.

The NIST CRMs 1573a tomato leaves, 1577c bovine liver, and 1566b oyster tissue (NIST, Gaithersburg, Maryland, USA) were analyzed for validation of the MIIS method. Approximately 0.2 g of each CRM was digested in 7.0 mL of concentrated trace metal grade nitric acid (70% vol/vol) using a Discover SP-D 80 single reaction chamber microwave-assisted digestion system (CEM Corporation, Matthews, North Carolina, USA). A digestion blank was prepared using the same volume of nitric acid but without any added solid. The sample digestion consisted of a 5 min ramp to 200 °C, a 5 min hold at 200 °C, and a cooldown period until the temperature inside of the digestion vessel returned to room temperature. The CRM and blank digestate solutions were diluted to a final volume of 100.0 mL using distilled deionized water (DDI) and then analyzed directly as the sample portions of solutions 1 and 2 without any additional dilution. The standard portion of solution 2 contained the CRM analyte stock diluted to a concentration of 5.00 μg L^–1^.

### Instrumentation

All measurements were made using an Agilent 8800 triple quadrupole ICP-MS (Santa Clara, California, USA); however, the instrument was operated solely in its single quadrupole detection mode for all experiments presented here. The sample introduction system was composed of an Agilent model SPS4 autosampler, a concentric pneumatic nebulizer, and a cyclonic spray chamber. The parameters used for the operation of the ICP are provided in Table 1. The instrument’s “fast pump” option (a pump rotation speed of 0.50 rps) was utilized for both the sample uptake delay and post-measurement rinse. The operational parameters were largely the instrument’s default values. All monitored isotope mass-to-charge (*m*/*z*) ratios are provided in Table 2. To ensure that all signals measured over the course of the proof-of-concept MIIS measurements were of a suitable intensity, no isotopes with natural abundances of less than 1% were selected.

**Table 1:** Parameters Used for the Operation of the ICP-MS

parameter	value
RF power	1.55 kW
plasma gas flow rate	15.0 L min^–1^
auxiliary gas flow rate	1.0 L min^–1^
carrier gas flow rate	1.05 L min^–1^
sampling depth	10 mm
pump speed	0.10 rps
uptake delay	45 s
stabilization time	30 s
rinse time	45 s
replicates	5
sweeps per replicate	50
integration time	0.100 s

**Table 2:** Monitored Species of Isotopes^a^

element	use	isotopes (*m*/*z*)
Li	analyte	6, 7*
Be	analyte	9
V	analyte	51
Cr	analyte	53
Mn	analyte	55
Co	analyte	59
Sr	analyte	86, 88*
Mo	analyte	92, 94, 95*, 96, 97, 98, 100
Ag	analyte	107*, 109
Cd	analyte	111*, 112, 113, 114, 116
Nd	internal standard	142, 143, 144, 145, 146*, 148, 150
Gd	internal standard	155, 156, 157*, 158, 160
Dy	internal standard	161, 162, 163*, 164
Er	internal standard	166*, 167, 168, 170
Yb	internal standard	171, 172*, 173, 174, 176
Tl	analyte	203, 205*
Pb	analyte	204, 206, 207, 208*
U	analyte	238

a For species with more than one selected isotope, the “best” selection as recommended by the instrument software is denoted by an asterisk.

### Matrix Effect Correction and Calibration Technique Comparison

The ability of MIIS to correct for matrix effects was evaluated by spiking 0.500 μg L^–1^ of the prepared analyte stock solution into three sample matrices, namely, DDI, 20% (v/v) ethanol, and 0.1% (m/v) calcium. The standard portion of solution 2 contained 5.00 μg L^–1^ analyte stock. MIIS results were compared to those obtained using EC, IS, SA, MISC, and MICal using the same sample spike concentration. Conventional EC and IS calibrations were carried out using a five-point curve, with analyte concentrations ranging from 0.00 to 5.00 μg L^–1^, prepared in 1% (v/v) trace metal grade HNO_3_ in DDI. Yb was incorporated into the solution preparation for the IS comparison at a concentration of 5.00 μg L^–1^. SA measurements used a four-point curve with added analyte concentrations ranging from 0.00 to 2.00 μg L^–1^. MICal and MISC were performed according to their respective literature procedures,^34,41^ with standard concentrations of 5.00 and 1.00 μg L^–1^, respectively. The internal standards used for the MISC measurement were the same as those used for MIIS (Nd, Gd, Dy, Er, and Yb) and were incorporated into the standard and sample solutions at a concentration of 5.00 μg L^–1^. Comparisons made using EC, IS, SA, and MISC employed only the ″best″ analyte isotope as recommended by the instrument software (and denoted in Table 2). The MICal comparison employed all suitable analyte isotopes identified for the MIIS measurement. All results were blank corrected by subtracting the concentration for each analyte determined in each simulated sample matrix prior to the addition of the analyte spike.

## 3 Results and Discussion

### Further Explanation of MIIS Plots

For a MIIS measurement, solutions 1 and 2 are prepared and analyzed as in any standard quantitative analytical procedure using typical instrument parameters and total measurement times. Internal standard signal ratios are calculated for each solution, just as in conventional internal standardization. However, monitoring multiple analytes and internal standard isotopes produces a high number of signal ratios that are used for the calibration. Consider the determination of Cd using Nd, Gd, Dy, Er, and Yb as internal standards. Using the monitored *m*/*z* values provided in Table 2 results in a total of 125 signal ratios for each solution, derived from five Cd isotopes and the 25 total isotopes measured across the five internal standards. According to eq 7, the signal ratios from solution 1 are plotted on the *y*-axis against their corresponding ratios measured in solution 2. A representative MIIS calibration plot for the determination of Cd in the oyster tissue CRM is shown in Figure 2. Taking the slope of the line, the 5.00 μg L^–1^ Cd standard concentration added to solution 2, and eq 8, the determined concentration of Cd in the oyster tissue digestate is 4.90 μg L^–1^. Accounting for the 0.2009 g mass of the CRM digested, the 100.0 mL final volume of the digestate solution, and the 5.00 mL volume of digestate used for the preparation of solutions 1 and 2 returns a Cd concentration of 2.44 μg g^–1^. This is a recovery of 98.4% of the expected value of 2.48 μg g^–1^ of Cd in the oyster tissue. Note that all calculated signal ratios from the various analyte and internal standard isotope combinations align into a single linear relationship.

**Figure 2:**
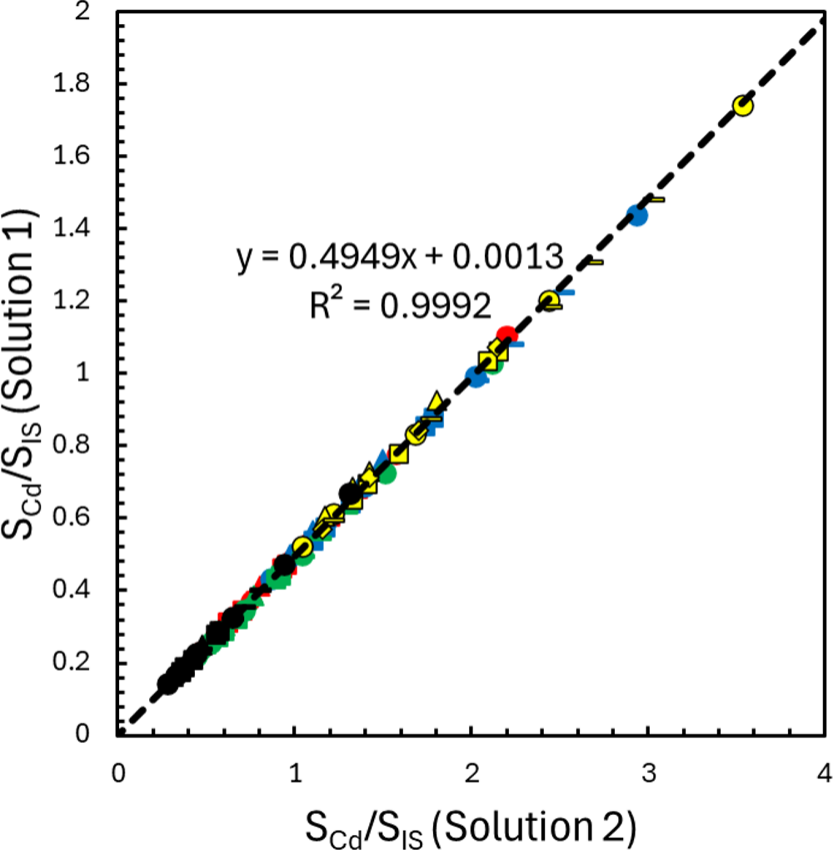
Representative MIIS calibration plot prepared for the determination of Cd in the oyster tissue-certified reference material. The red, blue, green, yellow, and black points correspond to the signal ratios obtained using the Cd isotopes at 111, 112, 113, 114, and 116 *m*/*z*, respectively. The circles, squares, triangles, diamonds, and dashes correspond to the signal ratios obtained using the internal standards Nd, Gd, Dy, Er, and Yb, respectively.

### Limits of Detection

Limits of detection (LODs) using MIIS were determined by analyzing 10 prepared pairs of solutions 1 and 2, using pure DDI as the sample portion of each. The concentration of the standard added to solution 2 was 5.00 μg L^–1^ for all analytes. LODs obtained using conventional EC are also provided. The values reported in Table 3 were calculated as three times the standard deviation of the 10 determined replicate blank concentrations. MIIS resulted in largely typical LODs achieved when using single quadrupole ICP-MS, on the order of single-digit ng L^–1^ for most analytes. These results are similar to those obtained using SDA, MCDA, and MICal for some of the same analytes under similar operational parameters using single quadrupole ICP-MS.^30,31,34,48^

**Table 3:** Limits of Detection Were Determined for a Suite of Analytes Using MIIS and EC

element	MIIS (ng L^–1^)	EC (ng L^–1^)
Li	10	8
Be	2	2
V	6	5
Mn	40	40
Co	2	2
Ag	40	30
Cd	6	4
Tl	2	2
Pb	10	9
U	0.7	0.5

### Working Range

A practical working range for MIIS was established by keeping the analyte standard concentration in solution 2 constant at 5.00 μg L^–1^ but altering the concentration of the sample spiked into the two solutions from 50.00 ng L^–1^ to 50.00 μg L^–1^. DDI was used as the sample matrix for determining the working range. To maintain visual clarity, results for V, Co, Cd, and Tl are presented in Figure 3, but similar trends were observed for all monitored analytes. Results for every selected analyte are provided in Table S1 in the Supporting Information. In general, a single standard concentration was sufficient to accurately quantify analyte concentrations from the method’s detection limit up to levels approximately 10 times higher than the standard added to solution 2, covering a range approaching four orders of magnitude. Across this range, analytes were determined within a few percent of the spike level with relative standard deviations on the order of a few percent. As one would expect, measurement precision worsens slightly as sample concentrations decrease toward the LOD. A decline in precision also occurred at the higher end of the tested sample concentration range when analyte levels began to exceed the added standard concentration. This result was also anticipated, as distinguishing a small concentration of standard from a large sample concentration (as both are present in solution 2) becomes increasingly difficult the more drastic the concentration difference becomes. This limitation is not specific to MIIS, with similar results noted when using other multisignal calibration methods.^20,33,47^

**Figure 3:**
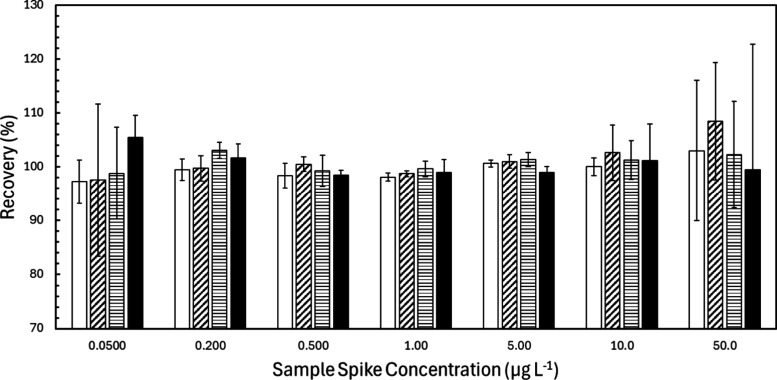
Analyte percent recoveries were obtained when purposefully varying the concentration of a suite of analytes spiked into a sample. The standard concentration was kept constant at 5.00 μg L^–1^. Results are reported as the average recovery for three MIIS replicates, with error bars denoting plus or minus one standard deviation. The white, diagonal striped, horizontal striped, and black bars correspond to analytes V, Co, Cd, and Tl, respectively.

### Certified Reference Materials

The proposed MIIS method was validated by determining trace analytes in NIST-certified reference materials (CRMs) 1573a tomato leaves, 1577c bovine liver, and 1566b oyster tissue. The CRM digestate solutions were utilized as the sample in solutions 1 and 2 directly without further dilution. The standard component of solution 2 contained 5.00 μg L^–1^ of each analyte of interest. Results for triplicate MIIS analyses of the CRMs are listed in Table 4. The method provided impressive results, with analyte recoveries consistently near 100% and relative standard deviations largely on the order of a few percent. These results further exhibit the robustness of the MIIS approach, which enables accurate and precise quantification of a wide range of trace elements in diverse sample matrices using minimal solution preparation. Entries missing from Table 4 include three different scenarios. First, in the case of Ag in tomato leaves and V, Cr, and Ag in bovine liver, the expected concentration in solution after digestion was below the method’s limit of quantification, so these results were excluded from the table. On the other hand, Mn and Sr in the tomato leaves were present at concentrations well beyond the upper limit of the recommended working range detailed above (approximately 100 and 50 times higher than the added standard concentration, respectively), so these results were also excluded. Finally, information about U in bovine liver and Cr and Mo in oyster tissue was not included on the certificates.

**Table 4:** Analyte Concentrations and Percent Recoveries Were Determined for a Suite of Analytes in Certified Reference Materials^a^

element	tomato leaves			bovine liver			oyster tissue		
	certified (μg g^–1^)	found (μg g^–1^)	recovery (%)	certified (μg g^–1^)	found (μg g^–1^)	recovery (%)	certified (μg g^–1^)	found (μg g^–1^)	recovery (%)
V	0.835 ± 0.034	0.711 ± 0.020	85.2 ± 2.4				0.577 ± 0.023	0.558 ± 0.010	96.6 ± 1.8
Cr	1.988 ± 0.034	1.80 ± 0.12	90.5 ± 6.2						
Mn				10.46 ± 0.47	9.2 ± 1.3	88 ± 12	18.5 ± 0.2	16.6 ± 2.1	90 ± 13
Co	0.5773 ± 0.0071	0.643 ± 0.034	111.5 ± 5.8	0.300 ± 0.018	0.2867 ± 0.0056	95.6 ± 1.9	0.371 ± 0.009	0.3614 ± 0.0080	97.4 ± 2.2
Sr				0.0953 ± 0.0042	0.0935 ± 0.0034	98.2 ± 3.6	6.8 ± 0.2*	6.51 ± 0.11	95.7 ± 1.7
Mo	0.46*	0.492 ± 0.044	107.0 ± 9.5	3.30 ± 0.13	3.20 ± 0.16	97.0 ± 4.9			
Ag							0.666 ± 0.009	0.652 ± 0.061	98.0 ± 9.2
Cd	1.517 ± 0.027	1.337 ± 0.028	88.2 ± 1.8	0.0970 ± 0.0014	0.0952 ± 0.0025	98.1 ± 2.6	2.48 ± 0.08	2.42 ± 0.16	97.6 ± 6.4
U	0.035*	0.0333 ± 0.0058	95 ± 17				0.2550 ± 0.0014*	0.2318 ± 0.0038	90.9 ± 1.5

a The results are reported as the average plus or minus one standard deviation for three MIIS replicates. Analytes with concentrations listed on the CRM certificates but not certified are marked with an asterisk.

### Correction of Matrix Effects and Calibration Technique Comparison

Due to the nature of the solution preparation required for MIIS, the method is expected to correct for sample matrix effects. The strength of this correction was investigated by spiking 0.500 μg L^–1^ of the selected analyte suite into three test matrices, including DDI (used to represent minimal matrix effects), 20% (v/v) ethanol, and 0.1% (m/v) Ca. The complex ethanol and calcium matrices were selected based on previous studies demonstrating their problematic nature for direct analysis by ICP-MS.^31,48^ Table S2 in the Supporting Information presents a comprehensive comparison of MIIS analysis to conventional EC, IS, and SA calibration approaches, as well as to the multisignal methods MISC and MICal, which have limitations that are partially addressed by MIIS. Mn and Co were determined in the blank calcium matrix at levels greatly exceeding single digit μg L^–1^ leading to poor results for all tested calibration methods; thus, these were excluded from the summary for clarity. MICal was performed for all analytes that had more than one naturally occurring isotope. To maintain visual clarity, results for only Cd and Ag are presented in Figure 4.

**Figure 4:**
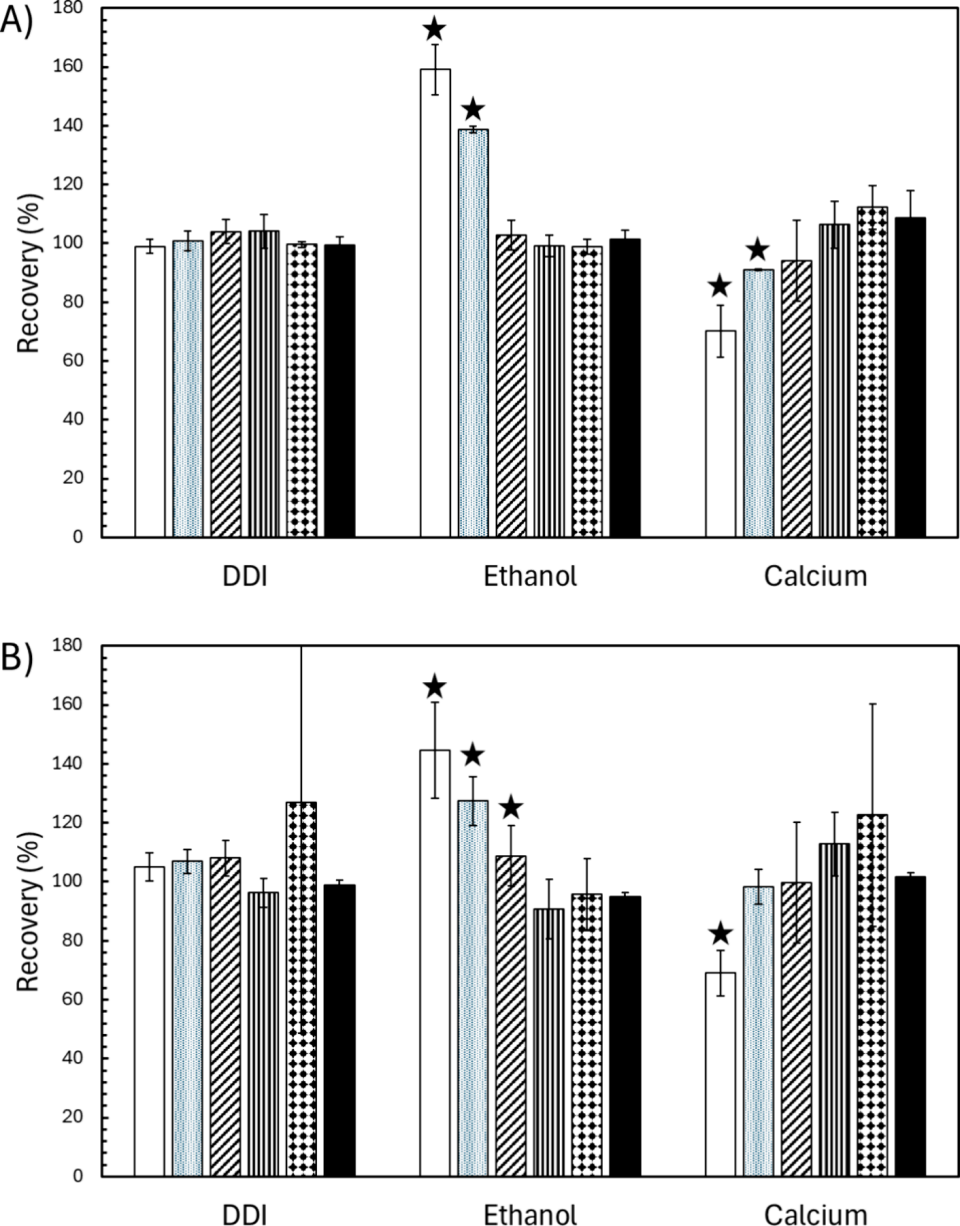
Analyte percent recoveries obtained for all tested calibration methods across the three sample matrices for Cd (A) and Ag (B). Results are reported as the average recovery for three replicates, with error bars denoting plus or minus one standard deviation. The white, gray, diagonal striped, vertical striped, dotted, and black bars correspond to EC, IS, MISC, SA, MICal, and MIIS, respectively. Results marked with a star were statistically different from the MIIS result in the same matrix at the 99% confidence level, as determined by single-factor ANOVA.

MIIS outperformed traditional EC and IS, as well as MISC, with those strategies producing analyte- and matrix-specific recoveries ranging from 70 to 222%. Single-factor ANOVA noted a statistical difference at the 99% confidence level for 16 of the EC results, 11 of the IS results, and 4 of the MISC results when compared to the 18 total MIIS results in the complex ethanol and calcium matrices, as noted in Table S2. The MIIS result was more accurate for all of these cases. In general, MIIS performed similarly to SA, as was expected, since both methods incorporate matrix matching into their solution preparation. Recoveries using both techniques were approximately 100% with relative standard deviations on the order of a few percent for all analytes tested. However, the reduced sample preparation and corresponding lessened measurement time result in MIIS offering significant practical advantages over SA. The MICal approach provided results comparable to MIIS, which was also expected. MICal can be applied only to analytes that have several measurable isotopes, a limitation that is eliminated when using MIIS. MICal was successful in determining Cd and Pb, which have both been exhibited elsewhere.^34–39^ MICal was attempted for Li, Ag, and Tl, which each have only two naturally occurring isotopes. MICal results obtained for Li and Tl were largely suitable, although the resulting calibration curves only had two points. In contrast, the MICal results obtained for Ag were very inconsistent. The isotopes of Ag have natural abundances of 51.8 and 48.2%, and thus, the points in the attempted MICal curve are very close together, resulting in what was essentially a one-point calibration curve and very poor precisions. Using MIIS as an alternative to MICal removes all doubt that poor MICal precisions are due to an insufficient number of calibration points through the creation of many internal standard ratios from a few analyte isotopes, allowing the technique to be reliably extended to any analyte element of interest with no regard needed for isotopic abundance.

### Use of Internal Standards and Isobaric Interferences

Internal standardization assumes that a chosen IS is not present in the sample. Moreover, any isobaric interference on the IS signal caused by the sample matrix during an ICP-MS measurement renders it unusable. These assumptions are certainly true for traditional IS and MISC experiments, as the sample and standard solutions are prepared separately, and each must contain the same added concentration of the internal standard for the correction to be successful. However, this constraint does not apply to MIIS due to the nature of sample preparation. Because both MIIS solutions contain the same amount of the same sample, any species already present in the sample may be used as an internal standard, so long as that species is not also being monitored as an analyte. An example of this is shown in Figure 5A, which displays a MIIS determination of Cd in the oyster tissue CRM using the isotopes of Ni, Sr, and Rb naturally present in the sample as the internal standard species. The solutions prepared for the measurement shown in Figure 5A utilized the analyte stock that was used for the proof-of-concept MIIS measurements instead of the CRM-specific analyte stock that contained Sr as an analyte. Using eq 8 and the added standard concentration of 5.00 μg L^–1^ Cd, the determined Cd concentration in the oyster tissue was 5.05 μg L^–1^. Accounting for the 0.2009 g mass of the CRM digested, the 100.0 mL final volume of the digestate solution, and the 5.00 mL volume of digestate used for the preparation of solutions 1 and 2 returns a Cd concentration of 2.51 μg g^–1^, a recovery of 101% of the expected value of 2.48 μg g^–1^ Cd in the oyster tissue according to the CRM certificate.

**Figure 5:**
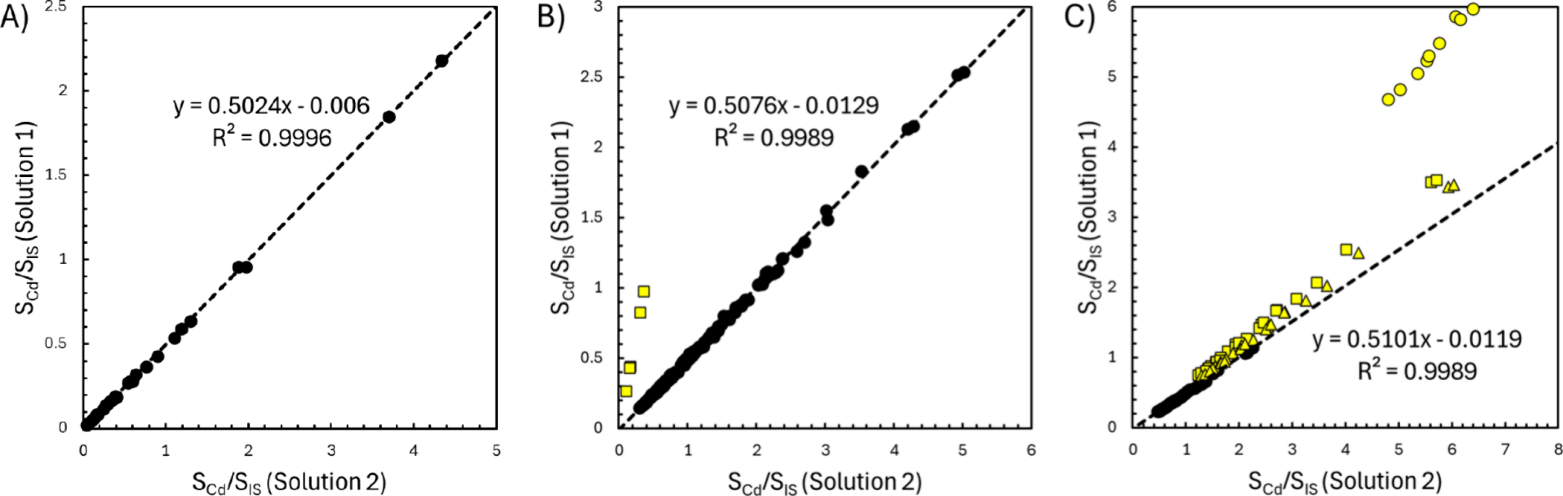
Representative MIIS plots exhibiting the selection of various internal standards and the simple identification of interferences. (A) Determination of Cd in the oyster tissue CRM using the Ni, Sr, and Rb present in the sample as internal standards. (B) Determination of Cd in the oyster tissue CRM using the selected internal standard suite as well as V. The black points represent the signal ratios obtained using the Cd signals and the Nd, Gd, Er, Dy, and Yb internal standards, with the yellow squares corresponding to the signal ratios obtained using V as an internal standard, which was present in the sample as well as in the standard portion of solution 2. (C) Determination of Cd in the oyster tissue CRM after spiking the sample with 50.0 μg L^–1^ of Sn. The black points correspond to the signal ratios obtained using the 111 and 113 *m*/*z* Cd isotopes, with the yellow squares, triangles, and circles representing ratios obtained using the 112, 114, and 116 *m*/*z* Cd isotopes, respectively, which suffer from isobaric overlap with Sn.

The restriction of the presence of the internal standards used in MIIS is not removed per se but simply altered in theory when compared with traditional IS and MISC measurements. All solutions in MIIS (as well as in IS and MISC) must contain identical amounts of the internal standard to ensure valid correction. In MIIS, the restriction is that any selected internal standard isotope must not be present in the standard aliquot added to solution 2, as that is what differentiates the two prepared solutions. This is demonstrated in Figure 5B, which shows the determination of Cd in the oyster tissue CRM from one of the triplicate MIIS measurements shown in Table 4. The line of best fit was generated using ratios calculated from the designated internal standard isotopes of Nd, Gd, Er, Dy, and Yb, represented by the black circles. The yellow squares, which fall clearly off the MIIS trendline, correspond to the signal ratios calculated when attempting to use V as an internal standard. As V was present both in the oyster tissue sample and in the standard portion of solution 2 (as it was being quantified as an analyte), its corresponding signal ratios do not follow the same trend as the true internal standards. This highlights the requirement that any internal standard selected for calibration by MIIS must not be introduced with the standard solution. Any such attempted internal standard falls obviously outside of the rest of the calibration plot and can be excluded from the calculation.

Isobaric overlaps on selected analyte isotopes from other species present in the sample can also bias the MIIS results. Monitoring multiple signals per analyte (if available) allows these outliers to be readily identified and excluded, just as in other MEC and MICal style measurements.^33,34,40,47^ This is exhibited in Figure 5C, which displays the determination of Cd in the oyster tissue CRM that was purposefully spiked with 50.0 μg L^–1^ of Sn. The line of best fit was generated using the black points, corresponding to the signal ratios calculated for the Cd 111 and 113 *m*/*z* isotopes (which are free from overlaps with Sn) using Nd, Gd, Er, Dy, and Yb as internal standards. The yellow squares, triangles, and circles correspond to the 112 (24.1% abundance), 114 (28.7% abundance), and 116 (7.49% abundance) *m*/*z* Cd isotopes, respectively, which have isobaric overlaps with the 112 (1.0% abundance), 114 (0.7% abundance), and 116 (14.5% abundance) *m*/*z* Sn isotopes. The deviation from the linear relationship caused by the Sn interference is easily identifiable, allowing the calculation to be performed using only the isotopes that are interference free.

### Effects of the Number of Isotopes Monitored

In an effort to investigate how measurement accuracy and precision changed as a function of the species monitored during an experiment, MIIS calculations were performed using various combinations of the selected analyte and internal standard isotopes, ranging from all monitored isotopes to only the “best” isotopes as recommended by the instrument’s software, and by utilizing only one individual internal standard species. It is important to note that there were some isobaric overlaps between the internal standards, occurring at *m*/*z* values of 156 (20.5% Gd and 0.1% Dy), 158 (24.8% Gd and 0.1% Dy), 160 (21.9% Gd and 2.3% Dy), 162 (25.5% Dy and 0.1% Er), 164 (28.2% Dy and 1.6% Er), 168 (26.8% Er and 0.1% Yb), and 170 (14.9% Er and 3.0% Yb). In these cases, the *m*/*z* value was assigned to the IS species with a higher natural abundance, as indicated in Table 2. However, these overlaps did not affect any of the results because all of the IS species were present at equal concentrations in both solutions 1 and 2 and did not suffer overlap from any of the analytes, as detailed above in the discussion of IS restrictions in MIIS. The impact of the number and the nature of the isotopes used in the calculation on the accuracy and precision of MIIS determinations for Tl in the three simulated complex sample matrices is presented in Table 5. While the results correspond to the determination of Tl alone, identical trends were observed for all of the analytes shown in Table S2. Representative MIIS plots of three combinations of Tl and internal standard isotopes in different sample matrices are shown in Figure 6. The different isotopic combinations yielded curves for Tl ranging from a minimum of four to a maximum of 50 calibration points. Overall, the number of isotopes used in the calculation did not significantly affect the accuracy or precision of the results, with the 0.500 μg L^–1^ spike of Tl recovered to within a few percent for each of the tested sample matrices. The recoveries are largely indistinguishable from one another when the relative standard deviation of the triplicate measurements is taken into account for all of the matrices and various isotopic combinations. Single-factor ANOVA noted no statistical difference at the 99% confidence level between the results obtained when utilizing all of the monitored isotopes (consistent with Figures 2 and 6A, and the uppermost row of data in Table 5) and all of the other tested isotopic combinations for each sample matrix. All results presented elsewhere in this article utilized the full set of monitored analyte and internal standard isotopes, maximizing the number of points used in the calibration curve.

**Figure 6:**
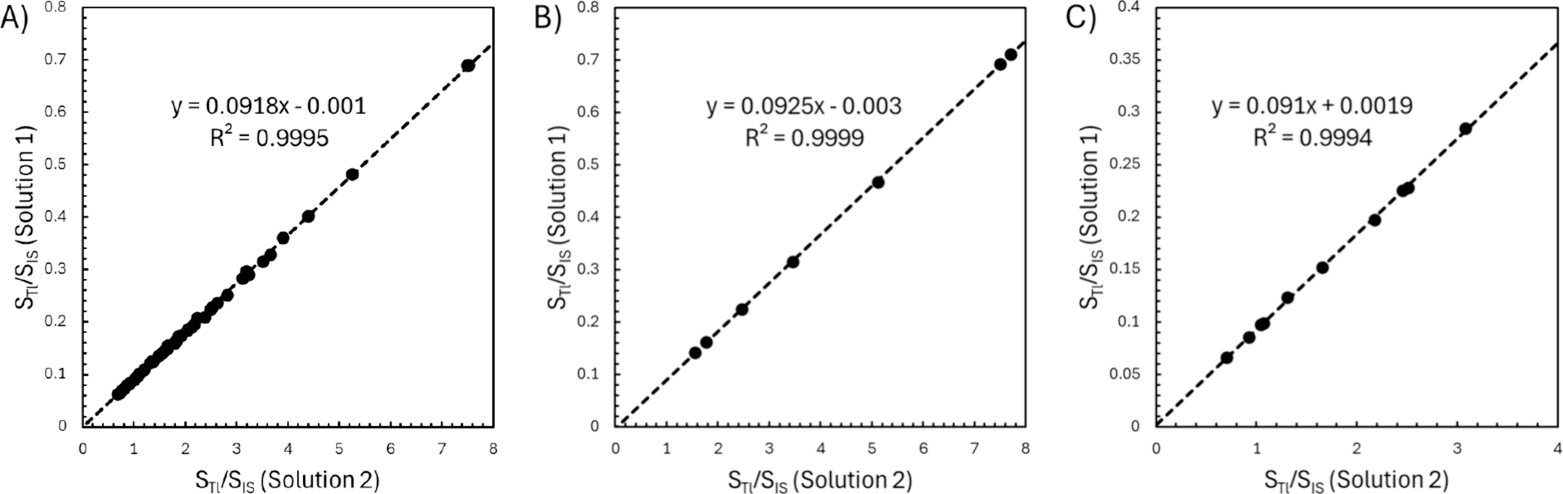
Representative MIIS plots obtained for the determination of a 0.500 μg L^–1^ spike of Tl into various sample matrices using different combinations of monitored isotopes. (A) In calcium, using both Tl isotopes and all of the monitored internal standard isotopes. (B) In ethanol, using the 205 *m*/*z* Tl isotope and only Nd isotopes as the internal standards. (C) In DDI, using both Tl isotopes and the highest-ranked isotope for each internal standard.

**Table 5:** Percent Recoveries Obtained for a Spike of 0.500 μg L^–1^ Tl in Various Sample Matrices Using Different Combinations of Monitored Isotopes^a^

Tl isotopes	IS isotopes	points in curve	DDI	ethanol	calcium
both	all	50	98.0 ± 0.9	101 ± 5	100 ± 6
	best for each	10	97 ± 2	101 ± 7	107 ± 7
	all Nd	14	98.1 ± 0.5	101 ± 4	103 ± 8
	all Gd	10	97 ± 3	101 ± 7	100 ± 7
	all Dy	8	97 ± 3	101 ± 8	99 ± 7
	all Er	8	97 ± 4	101 ± 6	100 ± 7
	all Yb	10	97 ± 3	101 ± 7	101 ± 7
best	all	25	98.4 ± 0.7	101 ± 4	103 ± 8
	best of each	5	100 ± 2	100 ± 6	104 ± 7
	all Nd	7	98.5 ± 0.7	101 ± 4	104 ± 9
	all Gd	5	100 ± 2	100 ± 5	102 ± 7
	all Dy	4	99 ± 3	100 ± 7	100 ± 10
	all Er	4	98 ± 5	101 ± 5	103 ± 8
	all Yb	5	98 ± 4	101 ± 6	102 ± 8

a The “best” isotopes for each species as recommended by the instrument’s software are marked in Table 2. The results are reported as the average recovery plus or minus one standard deviation for three MIIS replicates.

Recent investigations have detailed mechanisms by which ICP instrumentation can cause instrumental isotopic fractionation (IIF) resulting in inconsistent signal levels that can influence the precision of measurements, especially when determining isotopic ratios.^49,50^ The MIIS results presented here do not appear to be significantly impacted by IIF, as statistically similar results are obtained regardless of the identity of the internal standard elements and isotopes used. This could be attributed to the fact that each calibration point is created using the same internal standard isotope measured in each of the two solutions that have identical sample matrices, or potentially that minor fluctuations caused by IIF are “averaged out” in MIIS due to utilizing multiple species and isotopes, akin to the more encompassing internal standard effect noted in the original description of MISC.^41^ Additionally, monitoring as many viable isotopes as possible makes the identification and exclusion of potential interferences simpler, as detailed above. In the case that an interference is identified, all pertinent data would already be collected, and the isobaric overlap can simply be excluded from the calculation. Regardless, these findings highlight both the robustness and flexibility of the MIIS technique, as it remains effective even when limited to a single analyte isotope or a single internal standard species, an advantage in terms of practical applicability over the previously described MICal and MISC methods.^34,42^

## 4 Conclusions

Multi-isotope internal standardization (MIIS) is a matrix-matched calibration strategy specifically developed for use with ICP-MS that extends the principles of recently described multisignal calibration techniques by utilizing multiple analyte and internal standard isotopes to perform a calibration. While developed with ICP-MS in mind, the method could be extended to molecular mass spectrometry in theory as long as suitable internal standards free from analyte isobaric overlap are identified. The results presented here exhibit the strengths of the technique but also speak to its overall simplicity. While only requiring the preparation of two solutions, MIIS is capable of generating a multipoint, matrix-matched calibration curve with an extraordinarily high number of points, bypassing the need for traditional serial dilutions and extensive solution preparation.

MIIS demonstrated strong performance in both accuracy and precision for sample matrices known to be difficult for direct analysis by ICP-MS. Spike recovery experiments and CRM analyses yielded recoveries of approximately 100% with relative standard deviations on the order of a few percent. The method outperformed EC, IS, and MISC measurements in difficult matrices and provided significant practical advantages over traditional SA and the recently described MICal technique. MIIS effectively corrects for signal suppression or enhancement caused by complex sample matrices and allows for straightforward identification of isobaric interferences. Limits of detection and the working range for MIIS were similar to or slightly better than those obtained using comparable multisignal techniques, confirming that the simplicity of MIIS does not sacrifice analytical rigor. As such, MIIS represents a robust, high-throughput alternative for quantitative trace elemental analysis in complex sample matrices using ICP-MS.

## Supplementary Material





## Data Availability

All data will be made available upon request to the corresponding author. A representative set of MIIS data and example calculation for the determination of trace analytes in the bovine liver CRM is provided in the Supporting Information.

## Notes

Comprehensive comparison of all analytes in the working range experiments and calibration method comparison (PDF)
Example MIIS calculations for the bovine liver CRM (XLSX)
