# Supplementary material for: Multi-Isotope Internal Standardization for Inductively Coupled Plasma Mass Spectrometry
Source: ACS Omega. 2025 Nov 14;10(46):55692–703. doi: 10.1021/acsomega.5c07066 (PMC12658662; doi:10.1021/acsomega.5c07066)
Supplement: Supplementary file 1 [file ao5c07066_si_001.pdf]

Multi-Isotope Internal Standardization for Inductively Coupled Plasma Mass Spectrometry

Micah X. DeCourcey, Abigail J. Crossman and Willis B. Jones*

Department of Chemistry and Biochemistry, University of North Florida, Jacksonville, FL, USA 32224

*Corresponding Author. Email: w.jones@unf.edu

Table S1. Percent recoveries obtained when purposefully varying the concentration of a suite of analytes spiked into a sample. The standard concentration was kept constant at 5.00 µg L^{-1}. The results are reported as the average recovery plus or minus one standard deviation for three MIIS replicates. The results also include a calculated sensitivity ratio (m_A/m_{IS}) for Solution 1 relative to Solution 2 for each analyte isotope and internal standard isotope pair at each concentration level, calculated for each individual solution according to the two halves of Eq. 6 in the main manuscript body. The sensitivity ratios are reported as the percent recovery of the calculated ratio for Solution 1 relative to the average calculated ratio for Solution 2 in the experiment set (and vice versa), plus or minus one standard deviation. Due to the number of isotopes monitored, a total of 3000 sensitivity ratios were calculated for each spiked concentration of analyte, except for the 50.0 ng L^{-1} spike for which analytes below the LOQ were excluded.

Element	50.0 ng L^{-1}	200 ng L^{-1}	500 ng L^{-1}	1.00 µg L^{-1}	5.00 µg L^{-1}	10.0 µg L^{-1}	50.0 µg L^{-1}
Li	102 ± 2	100 ± 7	102 ± 4	99 ± 2	102 ± 1	102 ± 6	110 ± 40
Be	90 ± 2	97 ± 6	107 ± 5	100 ± 3	101 ± 3	102 ± 7	110 ± 10
V	97 ± 4	100 ± 2	98 ± 2	98.1 ± 0.7	100.6 ± 0.7	100 ± 2	100 ± 10
Mn	< LOQ	90 ± 10	101 ± 3	100 ± 1	100 ± 1	103 ± 7	110 ± 20
Co	100 ± 10	100 ± 2	101 ± 1	98.8 ± 0.6	101 ± 1	103 ± 5	110 ± 10
Ag	< LOQ	100 ± 10	99 ± 2	101 ± 3	99 ± 3	100 ± 6	90 ± 10
Cd	99 ± 9	103 ± 1	99 ± 3	100 ± 1	101 ± 1	101 ± 4	100 ± 10
Tl	106 ± 4	102 ± 3	98.5 ± 0.9	99 ± 2	99 ± 1	101 ± 7	100 ± 20
Pb	120 ± 20	100 ± 3	102 ± 2	99 ± 1	101.2 ± 0.9	101 ± 3	110 ± 10
U	95 ± 3	102 ± 4	103 ± 2	101.2 ± 0.5	107.5 ± 0.4	102 ± 3	110 ± 40
Calculated m_A/m_{IS}	100 ± 10	100 ± 6	100 ± 4	100 ± 3	100 ± 2	100 ± 2	100 ± 6

Table S2. Percent recoveries obtained for a suite of analytes spiked into three sample matrices at 0.500 µg L^{-1} using various calibration techniques. The results are reported as the average plus or minus one standard deviation for three replicates of each technique. Results marked with a caret (^) correspond to calibration results that are statistically different from the MIIS results in the same matrix at the 99% confidence level (ANOVA). MICal results marked with an asterisk (*) correspond to analytes that had three or fewer suitable isotopes monitored.

Matrix	Method	Li	Be	V	Mn	Co	Ag	Cd	Tl	Pb	U
DDI	EC	102 ± 1	102 ± 3	101.8 ± 0.9	99.7 ± 0.3	102.6 ± 0.5	105 ± 5	99 ± 2	104 ± 1^	102.0 ± 0.5	102.0 ± 0.9
	IS (Yb)	104 ± 2	104 ± 3	103.6 ± 0.7	101 ± 1	104 ± 1	107 ± 4	101 ± 3	105.6 ± 0.9^	103.8 ± 0.6	104 ± 2
	MISC	103 ± 2	99 ± 1	106 ± 3	116 ± 4^	102 ± 2	108 ±6	104 ± 4	101 ± 1	104 ± 3	102 ± 3
	SA	105 ± 2	99 ± 4	104 ± 1	99.9 ± 0.5	102.8 ± 0.8	96 ± 5	104 ± 6	104 ± 2	100 ± 2	100 ± 1^
	MICal	97 ± 4*					130 ± 80*	100 ± 1	97 ± 4*	100 ± 2	
	MIIS	102 ± 4	107 ± 5	98 ± 2	101 ± 3	101 ± 1	99 ±2	99 ± 3	98.5 ± 0.9	102 ± 2	103 ± 2
Ethanol	EC	103 ± 5	222 ± 3^	121 ± 5^	127 ± 4^	125 ± 5^	140 ± 10^	159 ± 5^	113 ± 3^	104 ± 6	114 ± 5^
	IS (Yb)	90.0 ± 0.6^	194 ± 2^	105.1 ± 0.7^	111 ± 2^	108.7 ± 0.6^	127 ± 6^	138.6 ± 0.8^	99 ± 2	91 ± 2^	99.6 ± 0.6
	MISC	99 ± 5	106 ± 4	116 ± 5^	128 ± 3^	109 ± 4	114 ± 7^	103 ± 5	101 ± 2	112 ± 5^	98 ± 4
	SA	97 ± 1	98.7 ± 0.2^	99 ± 2	97 ± 2	97 ± 2^	90 ± 10	99 ± 4	99.0 ± 0.7	98 ± 3	98 ± 3
	MICal	101 ± 1*					100 ± 10*	99 ± 3	103 ± 7*	102 ± 5	
	MIIS	101 ± 3	110 ± 4	99 ± 3	104 ± 3	103 ± 1	95 ± 1	101 ± 3	101 ± 5	102 ± 3	102 ± 2
Calcium	EC	70 ± 10^	70 ± 10^	70 ± 10^			69 ± 8^	70 ± 9^	75 ± 9^	70 ± 10^	81 ± 9^
	IS (Yb)	97 ± 3	87 ± 5^	95 ± 5			98 ± 6	90.9 ± 0.3^	99 ± 3	92.9 ± 0.4	107 ± 2^
	MISC	80 ± 20	90 ± 20	90 ± 20			100 ± 20	90 ± 10	90 ± 10	90 ± 20	90 ± 10
	SA	108 ± 8	108 ± 7	100 ± 10			110 ± 10	110 ± 8	107 ± 4	100 ± 10	106 ± 7
	MICal	104 ± 1*^					120 ± 40*	112 ± 7	94 ± 5*	110 ± 10	
	MIIS	100.8 ± 0.6	104 ± 2	110 ± 10			107 ± 9	109 ± 9	102 ± 8	100 ± 10	100 ± 4
